# Naturalism, tractability and the adaptive toolbox

**DOI:** 10.1007/s11229-019-02431-2

**Published:** 2019-10-30

**Authors:** Patricia Rich, Mark Blokpoel, Ronald de Haan, Maria Otworowska, Marieke Sweers, Todd Wareham, Iris van Rooij

**Affiliations:** 1 Department of Philosophy, University Hamburg, Hamburg, Germany; 2 Department of Philosophy, University Bayreuth, Bayreuth, Germany; 3 Donders Institute for Brain, Cognition, and Behaviour, Radboud University Nijmegen, Nijmegen, The Netherlands; 4 Institute for Logic, Language and Computation, University of Amsterdam, Amsterdam, The Netherlands; 5 Department of Artificial Intelligence, Radboud University Nijmegen, Nijmegen, The Netherlands; 6 Department of Computer Science, Memorial University of Newfoundland, St. John’s, Canada; 7 Donders Institute for Brain, Cognition, and Behaviour, Centre for Cognition, Radboud University Nijmegen, Montessorilaan 3, 6525 HR Nijmegen, The Netherlands

**Keywords:** Epistemic rationality, Ecological rationality, Adaptive toolbox theory, Intractability, NP-hard, Evolution, Heuristics, Naturalism, Instrumentalism

## Abstract

Many compelling examples have recently been provided in which people can achieve impressive epistemic success, e.g. draw highly accurate inferences, by using simple heuristics and very little information. This is possible by taking advantage of the features of the environment. The examples suggest an easy and appealing naturalization of rationality: on the one hand, people clearly *can* apply simple heuristics, and on the other hand, they intuitively *ought* do so when this brings them high accuracy at little cost.. The ‘ought-can’ principle is satisfied, and rationality is meaningfully normative. We show, however, that this naturalization program is endangered by a computational wrinkle in the adaptation process taken to be responsible for this heuristics-based (‘ecological’) rationality: for the adaptation process to guarantee even minimal rationality, it requires astronomical computational resources, making the problem intractable. We consider various plausible auxiliary assumptions in attempt to remove this obstacle, and show that they do not succeed; intractability is a robust property of adaptation. We discuss the implications of our findings for the project of naturalizing rationality.

## Introduction

Naturalists argue that we need theories of rationality that apply to actual people, and this means grounding our theories in empirical facts, for example about the cognitive capacities of humans. Kitcher contrasts the naturalist standard with the traditional one:

What grounds the claim that our favored logical principles are prescriptions for thought? What are the sources of these principles? Do such idealized recommendations really apply to us? A traditional response is to propose that they present conceptual truths about rationality, thereby formulating an ideal at which we aim. *For naturalists, however, such prescriptions must be grounded in facts about how systems like us could attain our epistemic goals in a world like ours.* Simply asserting that prescriptions unfold our concept of rationality will be beside the crucial point (Kitcher 1992, p. 63, emphasis ours).

We can break the naturalist’s task into two components, then: The empirical component involves identifying real-world strategies and defining real-world success.^1^ The normative component is to show how they are connected.^2^

Psychologists appear to have developed a research program—Ecological Rationality—which does exactly this; it studies how people achieve practical success in inference, choice, and beyond (see Gigerenzer and Selten 1999; and Gigerenzer et al. 2011, for overviews and many papers). Human goals are summarized in the slogan “fast, frugal, and accurate”: we want to correctly guess who will win and trust those who are trustworthy, but we also want to do so with minimal time and effort. This often means drawing a conclusion based on the available evidence—and often only a small subset thereof—rather than gathering more. Empirically, therefore, Ecological Rationality explains people’s everyday cognition in terms of “fast and frugal” heuristics. Here and throughout, we are concerned with cognitive inference heuristics as characterized by Chow (2014, p. 1009), i.e., cognitive procedures that need not be exact or optimal and can be expressed as computational steps or rules (not merely informal ‘rules of thumb’). These heuristics are said to be *fast* when they require little cognitive resources for their execution, and *frugal* when they use only a limited part of the available information for their execution. Examples of such easy-to-use strategies include expecting the famous player to win the tennis match (Scheibehenne and Bröder 2011 and trusting those who smile at us.

Normatively, Ecological Rationality uncovers the connection between simple heuristics and our goals; it doesn’t just explain what we do, but why we are largely successful. Our success has a lot to do with our ability to match heuristics to the environments in which they perform well, and it would not be ecologically rational to use the ‘wrong’ heuristic for the context. In fact, we are usually right when we assume that Serena Williams will beat Jane Doe and that the person happily offering help is no con artist. We are therefore rational, despite drawing quick conclusions based on one or two pieces of evidence, because the rules of thumb that we have applied will usually get the job done when applied to the problems and in the situations for which we typically apply them. Note that ecological rationality is therefore instrumental: the ultimate standard is pragmatic success by our own lights, so truth and coherence are only valuable insofar as they help us achieve our goals (see Over 2004, for further discussion). In particular, even our ecologically-rational use of heuristics will sometimes lead us to false conclusions; this must just happen infrequently enough and/or in sufficiently unimportant situations that it isn’t worth it to us to do things differently.

Ecological Rationality’s prominence has been driven by its ability to provide many compelling examples of success through the use of heuristics (see Goldstein and Gigerenzer 2002, for a well-known example). Perhaps the most striking examples are those in which simple heuristics appear to outperform classically-rational procedures such as weighing and averaging (see Gigerenzer and Goldstein 1996, for an example; and Gigerenzer and Brighton 2009, for an overview).^3^ Gigerenzer and Sturm (2012) use examples of this kind to argue for a limited naturalization of means-ends rationality which takes heuristics to be rational when used in environments for which they are highly accurate. Wherever one draws the line between rational and irrational, such examples greatly strengthen the case for ecological rationality as a true species of normative rationality and not just a consolation prize. We do not rely on heuristics simply because better strategies are impractical, but because they often allow us to make correct choices and inferences in our environment.

One might still wonder, of course, how far this explanatory strategy can take us. Why think that heuristics usually serve us well? Even if many heuristics can perform well, how could we manage to use the right ones in the right contexts (Wallin and Gärdenfors 2000)? The adaptationist backstory is important here. There is no guarantee that any particular application of a heuristic gets us the best result, or even a good one; our environment can change and our old strategies can fail in new contexts. Nonetheless, it makes sense that a long period of selection in a relatively stable environment would have endowed us with an “adaptive toolbox”^4^ of heuristics, and the ability to apply them adeptly. This would explain why our heuristic use would generally be successful, allowing us to do at least well enough to survive and in many cases much better.^5^

Ecological Rationality therefore looks like a very promising (base for a) naturalist project. On closer inspection, however, we find an important explanatory gap. Specifically, although it may be easy for a person to apply a particular simple heuristic, learning which heuristic from the adaptive toolbox to apply is not. Quite the opposite, in fact: evolving even an adequate adaptive toolbox is an intractable problem. This fact is independent of the type of adaptive process, so it holds for natural selection, learning, etc. as well as combinations thereof (cf. Otworowska et al. 2018, 2015). This does not mean that it is impossible that we did evolve an adaptive toolbox through truly extraordinary luck, but it does mean that the adaptive toolbox hypothesis itself is not supported, and the existence of adaptive toolboxes would not really be explained, by appeals to evolution, since we would not expect evolution to yield adaptive toolboxes.

This is a problem for several reasons. Importantly, we want to adjudicate between available explanations of human cognition, for example between Bayesian explanations and heuristics-based explanations. Bayesian computation is known to face an intractability challenge (Kwisthout et al. 2011), and it is furthermore intuitive to many that being an ideal Bayesian reasoner would be very difficult, perhaps impossible.^6^ The fact that Adaptive Toolbox turns out to face its own intractability challenge means that its apparent plausibility advantage was illusory (see also Otworowska et al. 2018). The clever application of heuristics and the use of Bayesian inferences could both produce rational behavior, but we do not know how either would have become possible for humans. This puts epistemic naturalists in an especially difficult position. Naturalists must ground normative assessments in an empirical understanding of how people make (or could make) inferences and so forth, but our current level of understanding is lower than we thought, and a real limiting factor.

Without understanding how far heuristics can take us, it is harder to succeed at the delicate balancing act between the ‘ought implies can’ principle and non-trivial normative standards. While naturalists have made everyone more conscious of the demands imposed by our normative theories, there is at the same time broad skepticism of naturalization projects that set the bar of rationality too low, blurring the distinction between what humans do and what we ought to do. Studying simple heuristics (seems to) ensure that we are studying strategies that people *can* implement, but epistemologists are interested in those strategies mainly insofar as we *should* use them (because they are effective means for achieving our ends). For the naturalist content to start with the available concrete examples of human heuristic use, two important questions loom large: not only why we should think that heuristics more generally *would* allow us to perform well enough to count as rational, but whether they already do so. The second, descriptive question has its own normative implications because consciously acquiring a new toolbox of heuristics would itself probably go beyond what humans could do. In other words, whether humans ought make widespread use of heuristics depends both on whether those heuristics could yield good results in theory and on whether humans could implement them successfully, and the latter may depend on whether that is what we in fact already do.

A major open problem in cognitive science—namely whether Bayesianism, Adaptive Toolbox, both, or neither^7^ are ‘true’ in the relevant sense—therefore turns out to matter a great deal for naturalists. We focus here on Adaptive Toolbox, and its relatively more surprising tractability challenge. In this paper, we show that this challenge is even more serious than has been recognized, by providing new results demonstrating the robustness of the intractability challenge. We wish to emphasize that our arguments do not show that the adaptive toolbox hypothesis is incorrect, but rather that it is crucially incomplete. In line with this, we include constructive proposals for addressing intractability, and thereby improving our understanding of cognition.

At this point we would like to help to situate our argument by acknowledging that Ecological Rationality and the Adaptive Toolbox hypothesis are most often encountered within philosophy in the context of the so-called “rationality wars.” This refers, broadly speaking, to the disagreement about whether or not human rationality should be characterized by classical, coherentist norms stemming from logic and probability theory. On the one hand, humans seem not to conform to those norms and hence appear irrational (see e.g. Kahneman et al. 1982; Kahneman and Tversky 1973; Tversky and Kahneman 1974, 1981, 1983), but on the other hand, it has been argued that this conclusion is an artifact of using the wrong norms (cf. Boudry et al. 2014; Brighton and Todd 2009; Gigerenzer 1996; Hertwig and Gigerenzer 1999; Polonioli 2014; Wallin 2013) or does not adequately motivate a normative or explanatory switch to so-called “bounded rationality” (see e.g. Chater et al. 2003; Grüne-Yanoff 2008). Proponents of Ecological Rationality (among others) have argued that the right norms are based on correspondence instead of coherence: rational beliefs and actions are those that lead to adaptive decisions in the world (see e.g. Arkes et al. 2016; Berg 2014b; Katsikopoulos 2009; Polonioli 2016; Rich 2016, 2018b, for discussion). Central questions have been the extent to which the debate in the literature is substantial, rather than terminological or rhetorical (notably Samuels et al. 2002) and whether the views are in fact compatible (see e.g. Berg and Gigerenzer 2006; Rich 2018a; Sturm 2012, 2019).

It is important to note that our focus is different from much of this literature in that we are fundamentally concerned with the *explanatory plausibility* of the adaptive toolbox theory—which for a naturalist is a critical basis for normative assessments—whereas much of the debate (especially in philosophy) has focused on the question of whether so-called “bounded” or “ecological” real-world rationality is true, *normative* rationality; we take an affirmative answer as a starting point. Furthermore, we do not attempt to give a general assessment of the empirical support for the adaptive toolbox hypothesis, but rather focus on whether tractability considerations support this theory over others. Tractability has long been one of many distinct issues at play in the rationality wars, since the intractability of classical rationality has been used to argue that it is not a relevant notion of rationality.^8^ From a descriptive perspective, tractability is taken to be an essential feature of any account of rationality.

One important consequence of our focus on explanation is that we are primarily concerned with the role of evolutionary arguments within the broader Ecological Rationality program, although this has not been the case in the rationality wars in general; that is, our focus is on the Adaptive Toolbox hypothesis rather than Ecological Rationality more broadly (see Footnote 4 as well as our earlier explanation of why the Adaptive Toolbox hypothesis is important). We wish to make clear that by advancing our specific critique, we in no way mean to cast doubt on the viability of Ecological Rationality in principle from a normative perspective, nor do we mean to question the fact that this research program has succeeded in placing many important observations (such as the relevance of decision and inference *processes*, the importance of contextual factors, and the limits of coherence criteria of rationality) at the center of the community’s attention (Rich 2016).

### Overview

The paper proceeds as follows. In Sect. 2, we introduce the computational problem of adapting toolboxes of heuristics to a given environment. We first explain the problem informally, followed by precise formulations of the key ideas. We illustrate how our formalism applies to concrete cases and reflect on the search space of adaptive toolboxes for environments of realistic sizes.

In Sect. 3 we present intractability analyses for the formalization developed in Sect. 2. We first explain the concepts and techniques used from computational complexity theory to formally prove intractability (). Next we present a series of complexity results that show that the intractability of ecologically rational toolboxes is quite robust. Several intuitive proposals for ensuring tractability are shown to fail. Notably, the conditions sufficient for ecological rationality are not sufficient for the tractability of the adaptation process, and the idea that the toolbox would have evolved along with the environment does not help.

Finally, we discuss the nature of the results, their applicability, and their implications in Sect. 4. We begin by summarizing the results and explaining the core of the problem. Next, we address the main objections that we anticipate, defending the assumptions we do make, and arguing that the intractability challenge is not easily dismissed. We also explain what we think can be done about it, however. With this on the table, we step back and discuss what the intractability challenge means for naturalists.

## Formalizing toolbox adaptation

According the Adaptive Toolbox theory, our minds are endowed with a toolbox consisting of fast and frugal heuristics tailored for different epistemic and pragmatic decision tasks. Over the last two decades, a wide variety of concrete heuristics have been proposed to explain not only inferences but also moral, economic (Berg 2014a), medical (Gigerenzer and Kurzenhaeuser 2005), and other pragmatic choices (see Ralph Hertwig and The ABC Research Group 2013, for many examples). (Gigerenzer and Sturm 2012, Table 1) provide a useful overview of the contents of the adaptive toolbox. Our analysis is meant to cover the vast majority of these heuristics, as we work with generic “fast and frugal trees.” As Sweers writes,

Gigerenzer et al. have proposed a list of heuristics, including the recognition heuristic, the fluency heuristic, Take The Best, tallying, satisficing, 1/N, default heuristic, tit-for-tat, imitate the majority and imitate the successful (Gigerenzer, 2008; Gigerenzer & Sturm, 2012). ...All heuristics of this list can be represented as fast-and-frugal trees, except for the fluency heuristic and tallying (2015, p. 63).

Rationality, on this account, is to be understood as the fit between the heuristics in the adaptive toolbox and the environment of adaptation. This fit is called *ecological rationality*, and it is proposed to be the natural consequence of processes of adaptation, such as biological evolution, cultural evolution, learning, development, or some mix thereof. We present in this section the formalization of the process of toolbox adaptation that will serve in our complexity analyses in Sect. 3.

### Computational-level theory

In broad lines, the formalization of the toolbox adaptation problem follows the one proposed by Otworowska et al. (2018), with minor modifications to increase generalizability (see also Sect. 3). The formalization takes the form of an input-output mapping, consistent with theories at Marr’s (1982) computational level. We first introduce an informal version of the theory, after which we detail the fully formalized theory. We would like to reiterate at this point that toolbox adaptation (and the formalization we present here) is agnostic as to the nature of the adaptation process, i.e., the adaptation may be realized through any number of processes, either individually or mixed.
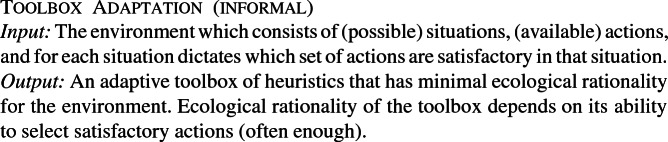


This input-output mapping captures informally what toolbox adaptation is considered to achieve, but it leaves out many specifics: What precisely is an adaptive toolbox? How can we determine ecological rationality? What is a situation? How are situations related to actions? The informal theory is underspecified, and this prevents a complexity analysis. We will build from this informal theory towards a fully formalized theory in a few more steps. The first step is to cast the incomplete informal theory in formal terms, after which we highlight and flesh out the missing parts.
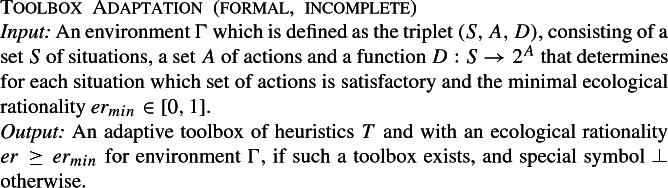


There are several parts that are still not fully formalized, namely, the toolbox of heuristics *T*, situations *S*, actions *A* and the ecological rationality *er* of a toolbox. Formalizing the toolbox *T* requires also formalizing situations *S* and actions *A*, so we address these simultaneously.

A toolbox of heuristics is characterized by two components: a set of heuristics and a selector heuristic that selects a heuristic to apply from that set. In this formalization we assume that a toolbox of heuristics consists only of one type of heuristic, a fast-and-frugal tree (Martignon et al. 2005; Luan et al. 2011; Martignon et al. 2008).^9^ A fast-and-frugal tree is a structure that can be formalized as follows:

#### Definition 2.1

**Fig. 1:**
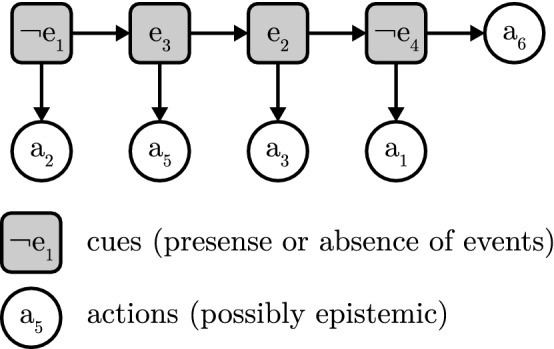
An abstract fast-and-frugal tree. Here, the arrows indicate the order of the cue-action pair sequence. The final action $a_6$ is the default action. These trees can be of arbitrary length and the formalism is agnostic as to the type of actions

A fast-and-frugal tree consists of an ordered sequence of cue-action pairs (*c*, *a*), where a cue *c* corresponds to an event *e* occurring or not occurring. The event *e* can be any of the events in the situations *S* in the environment $\Gamma$. Additionally, there is a default action $a_d$. Given a situation *s*, the fast-and-frugal tree selects the first action *c* for which the cue holds true in *s*. If no cue holds true, the default action $a_d$ is selected. Figure 1 illustrates an abstract fast-and-frugal tree.

**Fig. 2:**
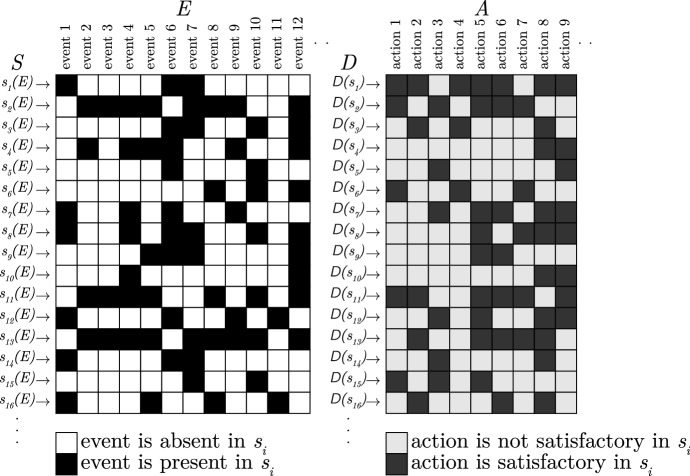
An abstract environment that represents the events *E*, situations *S*, actions *A* and in which situations which events are present (black square) or absent (white square) and which actions are satisfactory (dark gray) or not (light gray)

**Fig. 3:**
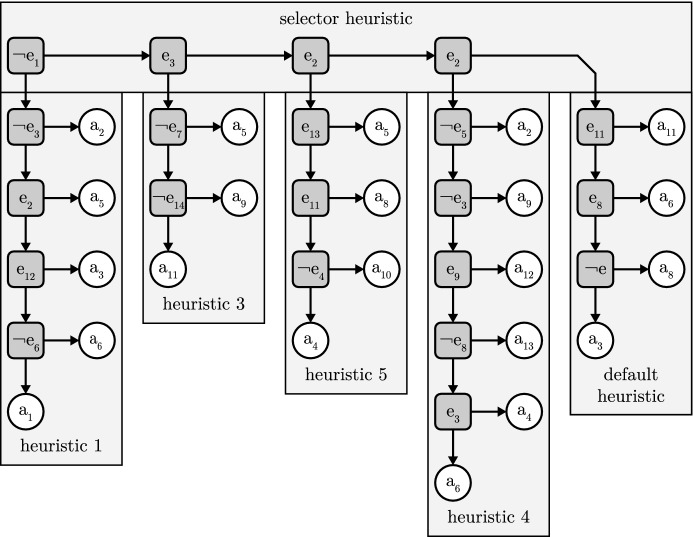
A toolbox of heuristics that consists of a selector heuristic and a collection of fast-and-frugal trees. Here, the selector heuristic selects a heuristic based on any of the four cues or if none of its cues evaluate to true it selects the default heuristic (far right)

The definition of a fast-and-frugal tree calls for a more detailed formalization of the environment, specifically, the addition that any situation $s\in S$ is defined by a the presence or absence of events. The environment $\Gamma$ is extended into a quadruple (*E*, *S*, *A*, *D*) by adding a set of events *E* that may occur in the environment and by associating to each situation $s_i \in S=\{s_1,\dots ,s_n\}$ a function $s_i:E\rightarrow \{true, false\}$ that specifies for each situation which events are present in that situation or not (Fig. 2). Note that, unlike the formalization of environments given in Otworowska et al. (2018), we allow multiple copies of a situation in *E*. We can now formalize a toolbox of heuristics (Fig. 3).

#### Definition 2.2

A toolbox of heuristics $T=(L,H)$ consists of a selector heuristic *L* and a set of heuristics $H=\{h_1,\dots ,h_m\}$, all of them are fast-and-frugal trees. The selector heuristic *L* is special in the sense that it consists of cue-heuristic pairs and a default heuristic, effectively ‘selecting’ a heuristic to use on the basis of present (or absent) events. Given a situation *s* (which is a truth-value assignment to events), a toolbox first uses the selector *L* to select a heuristic *H* on the basis of the available cues, and then uses *H* to select an action *a*, also on the basis of the available cues. We write *T*(*s*) to refer to the action *a* that is selected by *T* in situation *s*.

With a formalization of a toolbox of heuristics and the environment we can now also define the ecological rationality of a toolbox. The ecological rationality *er* specifies the fit between an adapted toolbox *T* and an environment $\Gamma =(E,S,A,D)$ as the proportion of satisfactory action selections:

$$\begin{aligned} er= & {} {\frac{\#~\text {situations where a satisfactory action is selected}}{{\#\,\mathrm {total\,number\,of\,situations}}}}\\= & {} \frac{|\left\{ s | s\in S ~\text {and}~ T(s)\in D(s)\right\} |}{|S|} \end{aligned}$$

With all of the details formalized, we can characterize toolbox adaptation formally. Note that we also introduce two additional parameters regarding the size of the toolbox, $\#h$ and |*h*|. These parameters limit the size of toolbox, thereby formalizing the notion of frugality.
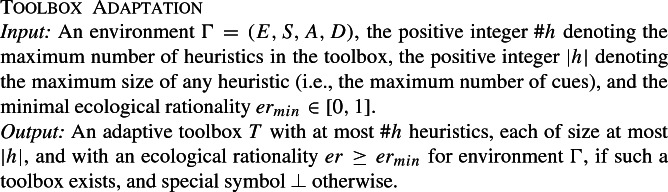

**Fig. 4:**
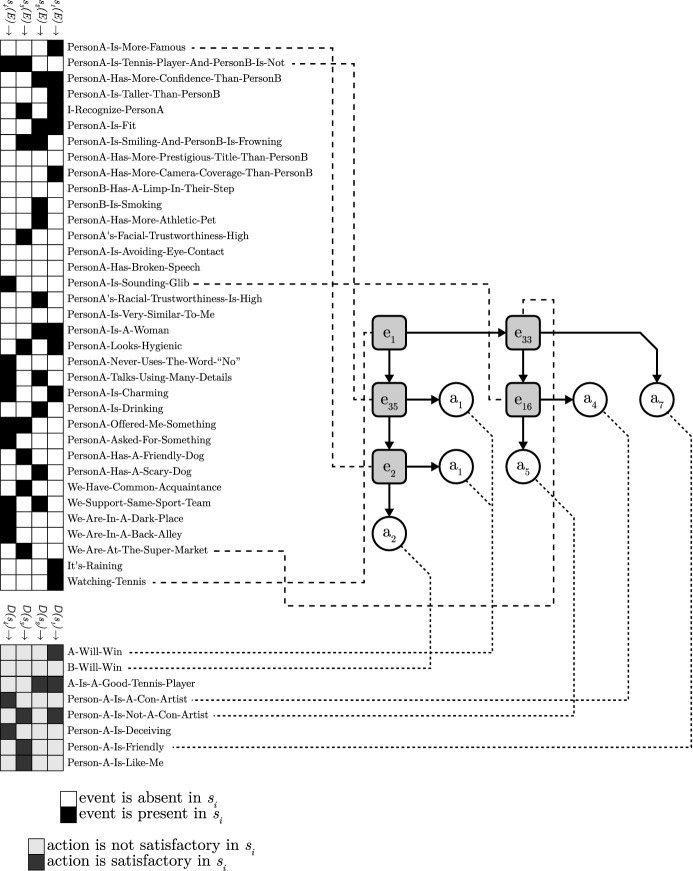
On the left is a toy example environment consisting of four situations. The first two columns correspond to the con-artist example from the Introduction, the third and fourth to the tennis example. On the right is an example toolbox, where cues are indicated by links to events and actions are indicated by links to the actions. The ecological rationality of this toolbox would be: $\frac{0+1+0+1}{4}=0.5$

**Fig. 5:**
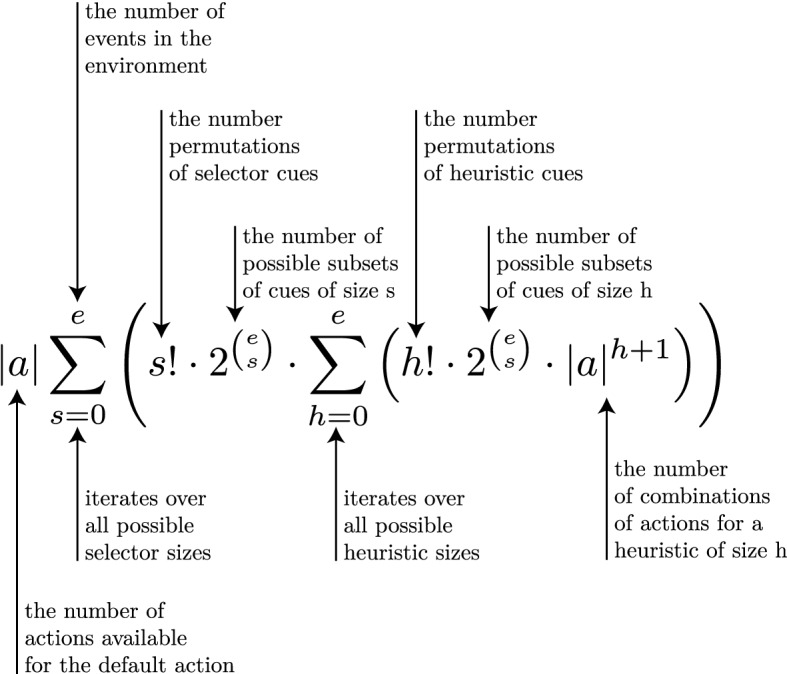
The formula describing how many toolboxes are possible for an environment with *e* events and *a* actions

**Table 1:** Illustration of the vast number of possible toolboxes for different environment sizes *e*, considering only 10 actions

Environment size *e*	Number of possible toolboxes	Scale example: Similar to the number of...
1	1080	
2	22,300	
3	334,520	
4	4,458,500	
5	56,109,040	
		...the number of neurons in a human brain $86\times 10^9$
		...the number of neuronal connections in a human brain $86\times 10^{14}$
10	$4.3 \times 10^{45}$	
20	$5.0 \times 10^{57}$	
30	$4.8 \times 10^{71}$	
40	$6.0 \times 10^{86}$	...elementary particles in the universe $10^{80}$
50	$2.2 \times 10^{103}$	...grains of sand in a universe entirely filled with sand $10^{95}$
60	$1.9 \times 10^{120}$	...neutrons in a universe entirely filled with neutrons $10^{128}$
70	$6.5 \times 10^{138}$	
80	$2.3 \times 10^{157}$	
90	$7.8 \times 10^{176}$	
100	$2.9 \times 10^{198}$	

We purposely presented the formalization of Toolbox Adaptation abstractly so as to show that the formalization is agnostic towards the encoding of events (these may correspond to complex or simple structures), the nature of the adaptation process (which may be realized by biological or cultural evolution, development, or otherwise), and can accommodate any structure in the environment (there are no constraints on the structure in $\Gamma$). We now turn back to the example heuristics from the introduction and see how they can be modeled using the formalization.

### Illustration

To give the reader better intuition of the formalization, we present a slice of an environment consistent with the examples from the Introduction in Fig. 4. It contains possible events that might occur in environment where we both adapted to deciding who will win a two-person competition (e.g. Serena or Jane) but also to decide whether or not to trust someone. In addition to possible events, the environment also dictates which actions are satisfactory in which situation. Hence, the formal environment contains a list of possible actions (or decisions) as well. These actions need not be physical; they can also be epistemic (i.e., belief revisions or inferences). The figure also shows a possible heuristic that has ecological rationality of 0.5 (i.e., it performs a satisfactory action 50% of the time). In a sense, the adaptation process has to figure out which events are relevant cues for the heuristic (the mappings indicated by the dashed lines, in the figure). Despite the fact that the final heuristic may use only a few cues (just 5 in the example), many different situation-cue mappings are possible.

Since empirical researchers consider specific toolboxes (and their application) and specific situations, the problem that is solved by the adaptation process remains invisible. However, when one considers the Adaptive Toolbox hypothesis itself, one has to face the fact that in principle many toolboxes are available, and an adequate one would need to be found. The number of possible toolboxes is directly related to the number of actions and events in the environment and grows more than exponentially (as Fig. 5 shows). Even for small environments and few available actions, the size of the search space of possible toolboxes is prohibitively big (see Table 1 for an example).

Although the size of the search space is titanic, its size alone does not prove it cannot efficiently be searched. In fact, some exponentially large search spaces can be efficiently traversed.^10^ However, as we will see, this is not the case for Toolbox Adaptation. To formally prove this, we will need to use computational complexity analysis.

## Computational complexity analyses

### Primer on computational complexity

We briefly introduce the basic concepts and proof techniques from computational complexity theory used in the complexity analyses of the Toolbox Adaptation problem (in Sect. 3.2.). For more details, we refer to textbooks on the topic, e.g. (Arora and Barak 2009; van Rooij et al. 2019).

Computational complexity theory is concerned with the mathematical study of the computational resources (time, space, randomness, etc.) consumed by computation. Solving some computational problems (i.e., input-output mappings) inherently requires such astronomical amounts of resources that we say the problems are ‘computationally intractable.’ Computational complexity theory provides analytic tools for answering the question: does there exist *any* tractable algorithm for a given problem *Q*? For this purpose, a distinction is traditionally made between *polynomial-time* algorithms (which run in time on the order of $n^c$, where *n* is a measure of the input size and *c* is some constant) and *non-polynomial* algorithms (which run in a time that cannot be upper-bounded by any polynomial). An example of non-polynomial time is *exponential* time (time on the order of $c^n$). As Garey and Johnson (1979) remark:

Most exponential time algorithms are merely variations on exhaustive search, whereas polynomial time algorithms generally are made possible only through the gain of some deeper insight into the nature of the problem. There is wide agreement that a problem has not been “well-solved” until a polynomial time algorithm is known for it. Hence, we shall refer to a problem as intractable, if it is so hard that no polynomial time algorithm can possibly solve it (p. 8).

To see why exponential time algorithms are traditionally considered intractable note that even for an input size of $n = 60$, an exponential running time of $2^n$ takes around $10^{20}$ steps. At 1000 steps per second, this would still take around $10^{17}$ seconds, about the lifespan of the universe. The input size for real world problems—which our adaptive toolbox is meant to allow us to solve—cannot generally be assumed to be small. As Gigerenzer and colleagues have put it:

The computations postulated by a model of cognition need to be tractable in the real world in which people live, not only in the small world of an experiment with only a few cues. This eliminates ${\mathsf {NP}}$-hard models that lead to computational explosion, such as probabilistic inference using Bayesian belief networks (Cooper, 1990), including its approximations (Dagum & Luby, 1993) (Gigerenzer et al. 2008, p. 263, references theirs).

Computational complexity analyses are typically performed with so-called *decision problems*, where the output of the problem is “yes” or “no” (1 or 0, respectively). The complexity class ${\mathsf {P}}$ consists of all such problems that can be solved in polynomial time. The Toolbox Adaptation problem introduced in Sect. 2, in contrast, is a so-called *search problem*; its output is a toolbox with minimal ecological rationality (if one exists). Its decision version instead asks *whether* a minimally ecological rational toolbox exists for a given environment. For complexity purposes, the two versions are closely related: if the decision problem is not in the class ${\mathsf {P}}$, then the search variant is not polynomial-time computable either. Some of the formal proofs in the Appendix work with the decision versions, but we will discuss the results and their implications in terms of the search versions for presentational purposes. We do need to consider decision problems a bit longer, in this section, to explain the key proof tools.

Computational complexity offers tools to assess whether or not a problem is tractably (polynomial-time) computable. The most commonly used tool for this is the complexity class ${\mathsf {NP}}$. Intuitively, the class ${\mathsf {NP}}$ contains all decision problems that are polynomial-time solvable, but likely also problems that are harder. Informally, a decision problem is in the class ${\mathsf {NP}}$ if for every yes-input there exists a solution that can be verified in polynomial time—and if for every no-input, no such solution exists. Formally, a decision problem is in ${\mathsf {NP}}$ if (i) there exists a polynomial function $p : \mathbb {N} \rightarrow \mathbb {N}$ and (ii) a polynomial-time algorithm *V* (the *verifier*) such that: for every input *x* it is the case that the output for *x* is 1 (“yes”) if and only if there exists a solution *y* of size *p*(|*x*|) such that *V* returns 1 (“yes”) when given *x* and *y* as input. It is an open problem whether ${\mathsf {P}}= {\mathsf {NP}}$, but it is widely believed in the field of theoretical computer science that ${\mathsf {P}}\ne {\mathsf {NP}}$ (Fortnow 2009). Neither we nor proponents of Adaptive Toolbox reject this common assumption (Martignon and Schmitt 1999; Schmitt and Martignon 2006), so we assume here that ${\mathsf {P}}\ne {\mathsf {NP}}$, and interpret our results accordingly.

To prove that a problem is intractable (not polynomial-time solvable), the notion of ${\mathsf {NP}}$*-hardness* is used. This notion is based on problem reductions. Intuitively, a reduction from a problem $Q_1$ to another problem $Q_2$ shows that $Q_2$ is at least as hard as $Q_1$: one can solve $Q_1$ by carrying out this reduction and then solving $Q_2$. The typical type of reductions that are used is that of *polynomial-time many-to-one reductions*. A (polynomial-time many-to-one) reduction from a problem $Q_1$ to a problem $Q_2$ is a function *R* that takes an input $x_1$ of $Q_1$ and produces an input $x_2$ of $Q_2$ and that satisfies the following conditions: (1) *R* is computable in polynomial-time, and (2) $x_1$ is a yes-input of $Q_1$ if and only if $x_2$ is a yes-input of $Q_2$.

**Fig. 6:**
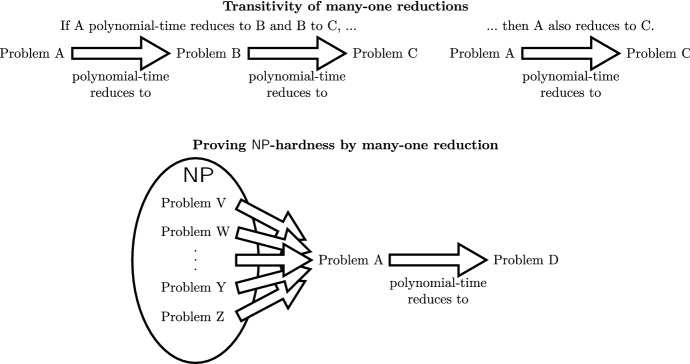
An illustration of the transitive relationship of many-one reductions and how these can be used to prove , i.e., by showing that all problems in  reduce to a problem of interest (here *D*)

A decision problem *Q* is said to be ${\mathsf {NP}}$*-hard*, then, if there exists a reduction from every problem in the complexity class ${\mathsf {NP}}$ to *Q*. Courtesy of the transitivity of many-one reductions (i.e., if decision problem *A* reduces to problem *B* and *B* reduces to *C* then *A* reduces to *C*), we can also show by reducing a known problem to a new problem of interest (see Fig. 6). Intuitively, ${\mathsf {NP}}$-hardness means that a problem is as hard as any problem in ${\mathsf {NP}}$. It holds that an ${\mathsf {NP}}$-hard problem is not solvable in polynomial time, unless ${\mathsf {P}}= {\mathsf {NP}}$—in other words, assuming that ${\mathsf {P}}\ne {\mathsf {NP}}$, an ${\mathsf {NP}}$-hard problem is not solvable in polynomial time. A problem is said to be ${\mathsf {NP}}$*-complete* if it is both in ${\mathsf {NP}}$ and ${\mathsf {NP}}$-hard.

Another type of reduction that is often used is that of (polynomial-time) Turing reductions. A *polynomial-time Turing reduction* from a problem $Q_1$ to a problem $Q_2$ is a polynomial-time algorithm that solves $Q_1$ and that has access to an oracle for $Q_2$. An *oracle* for $Q_2$ is a special (imaginary) device that, when given an input *x* of $Q_2$, returns the correct answer in a single computational step. We say that a problem *Q* is ${\mathsf {NP}}$*-hard under Turing reductions* if there exists a polynomial-time Turing reduction from every problem in the complexity class ${\mathsf {NP}}$ to *Q*. Courtesy of the transitivity of Turing reductions, one can also show such by reducing from a problem that is known to be under Turing reductions. Problems that are ${\mathsf {NP}}$-hard under Turing reductions are also not solvable in polynomial-time, unless ${\mathsf {P}}= {\mathsf {NP}}$.

We realize that the notion of reduction, of either type, can be tricky to understand at first. To support intuitive understanding of our proof arguments we will include graphical illustrations in the next section.

### Intractability results

We present here a set of complexity theoretic results on the intractability of the toolbox adaptation process. We probe the robustness of this intractability by analyzing various refinements of the computational-level characterization of Toolbox Adaptation in Sect. 2. Given that, we build on the original intractability () proof provided by Otworowska et al. (2018)—in part by exploiting the ability of our environments (noted in Sect. 2) to have multiple copies of the same situation. We start by restating the main theorem in Otworowska et al. (2018) below.

#### Theorem 3.1

(Otworowska et al. 2018) Toolbox Adaptation is , even when restricted to an arbitrary fixed $er_{min} \in (0,1]$.

#### Proof (sketch)

**Fig. 7:**
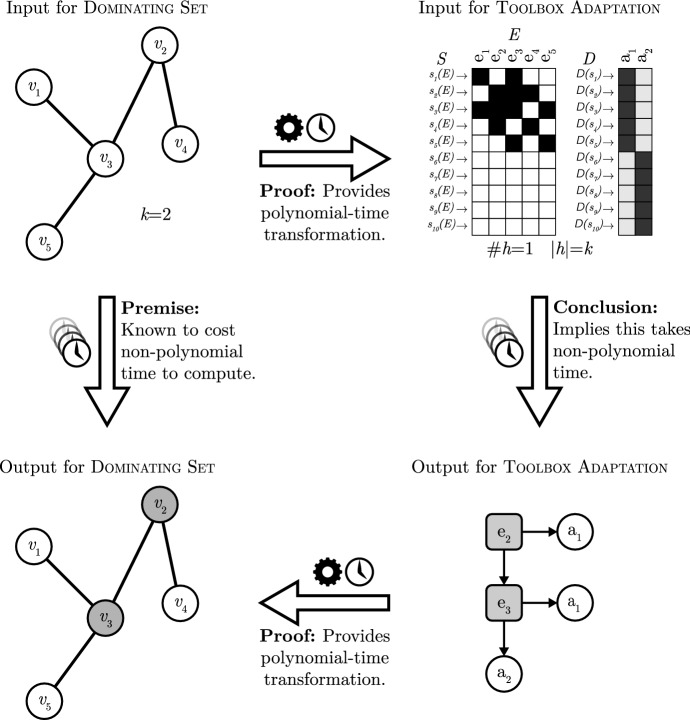
An illustration of the proof that Toolbox Adaptation is . Working from the premise that Dominating Set is (and therefore cannot be solved in polynomial time, unless ${\mathsf {P}}= {\mathsf {NP}}$), we construct two polynomial-time transformations. One transforms the input for Dominating Set to an input for Toolbox Adaptation, and the other transforms the output of Toolbox Adaptation to an output for Dominating Set. As said, these transformations run in polynomial time and hence we can conclude by contradiction that Toolbox Adaptation cannot run in polynomial time, otherwise one could solve Dominating Set in polynomial time (viz. by transforming the input for Dominating Set, then solving Toolbox Adaptation and then transforming the output back to Dominating Set). Here, the problem Dominating Set is a graph problem that asks whether or not there exists a subset of vertices (maximally of size *k*) that dominate (are a neighbour of) all other vertices in the graph

The original proof by Otworowska et al. (2018) is by reduction from the known ${\mathsf {NP}}$-hard graph problem Dominating Set (Garey and Johnson 1979). We provide an alternative (simplified) proof in the Appendix. This proof also shows ${\mathsf {NP}}$-hardness by giving a polynomial-time reduction from Dominating Set. See Fig. 7 for an illustration. $\square$

Theorem 3.1 demonstrates that there cannot exist any polynomial-time algorithm that, given an instance of the Toolbox Adaptation problem, can reliably produce a toolbox with minimal ecological rationality $er_{min}$ (for any $er_{min} > 0$) (as always, assuming that ${\mathsf {P}}\ne {\mathsf {NP}}$). This result is limited, however, in that it only demonstrates relative to the set of all logically possible environments, which may include many environments for which no ecologically rational toolbox exists. It is thus of interest to investigate whether toolbox adaptation is tractable if we restrict the domain of environments to only those affording ecologically rational toolboxes. For this purpose we consider a variant of Toolbox Adaptation where it is promised, for each environment, that there exists at least one (and possibly more) ecologically rational toolboxes. This type of problem variant is also known as a *promise problem*. Theorem 3.2 below shows that the ‘promise’ does nothing to make adaptation more tractable.

#### Theorem 3.2

Toolbox Adaptation is ${\mathsf {NP}}$-hard (under Turing reductions) even when a promise is given that there exists an ecologically rational toolbox for the given environment (of the right size).

#### Proof (sketch)

The general idea of the proof is the following. Suppose that a polynomial-time tractable algorithm *A* exists that computes an ecologically rational toolbox in situations where it is promised that such a toolbox exists. We show that we can then solve the ${\mathsf {NP}}$-complete problem of Satisfiability in polynomial time.

Since Toolbox Adaptation is ${\mathsf {NP}}$-hard, we know that we can reduce the ${\mathsf {NP}}$-complete problem Satisfiability to it (call the reduction *R*). Moreover, we can do this in such a way that a valid solution for Toolbox Adaptation can be efficiently translated to a valid solution for Satisfiability. To compute the answer for an input *x* of Satisfiability, we do the following. We firstly transform the input *x* to an input of Toolbox Adaptation by using the reduction *R*. Then we apply the algorithm for the promise variant of Toolbox Adaptation to *R*(*x*).

Then one of several situations is the case: either (i) the input *R*(*x*) fulfils the promise, and there exists an ecologically rational toolbox (of the right size), or (ii) no such ecologically rational toolbox exists. In case (i), the algorithm produces a solution, which can be transformed to a solution for the input *x* of Satisfiability. By efficiently verifying whether this solution for Satisfiability is valid, we are able to ascertain that the answer for *x* is ‘yes.’ In case (ii), the algorithm either (ii.a) produces a toolbox that is not ecologically rational (or not of the right size), or (ii.b) produces uninterpretable output. In case (ii.a), we can efficiently verify that the toolbox is not ecologically rational (or too large), and thus that the answer for *x* is ‘no.’ In case (ii.b), we also know that the output for *x* is ‘no.’ Thus, in all cases we solve the problem of Satisfiability correctly and efficiently.

**Fig. 8:**
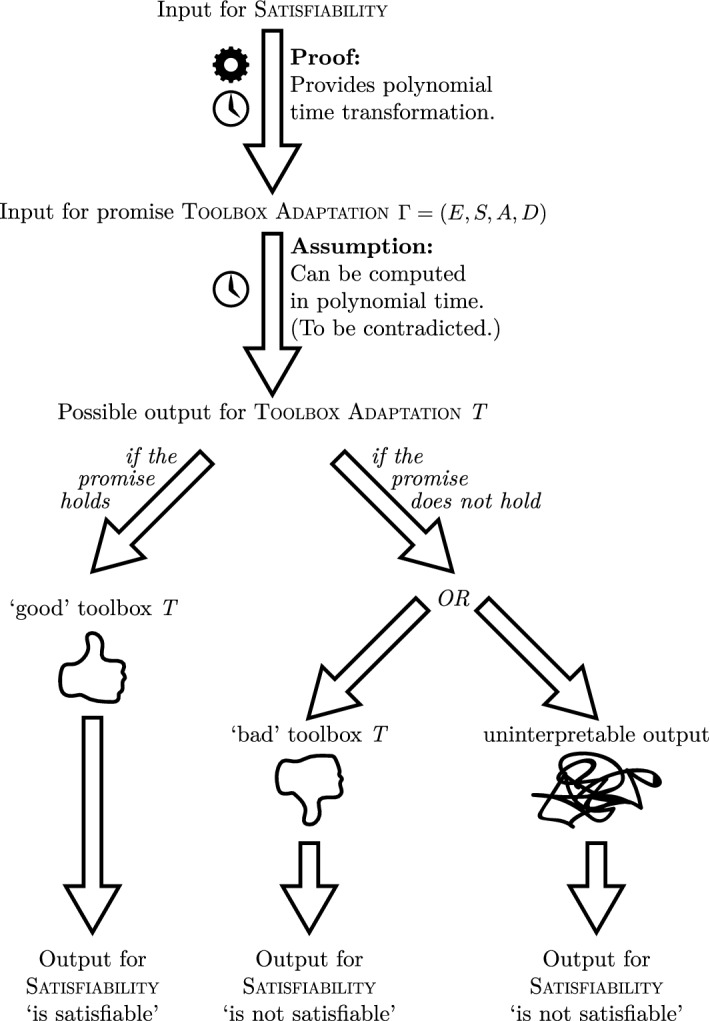
Illustration of the proof by contradiction that promise-Toolbox Adaptation is . Assuming that promise-Toolbox Adaptation can be computed in polynomial time, we show that one can solve Satisfiability in polynomial time. We transform the input for Satisfiability into an input for promise-Toolbox Adaptation, then apply the polynomial time algorithm to solve that instance, and using the schema illustrated here infer the solution for Satisfiability in polynomial time. However, since it is known that Satisfiability is , this schema cannot exist (assuming ${\mathsf {P}}\ne {\mathsf {NP}}$) and we reject our assumption that promise-Toolbox Adaptation can be solved in polynomial time

This procedure can be seen as a Turing reduction from Satisfiability to the promise variant of Toolbox Adaptation, and thus the problem is ${\mathsf {NP}}$-hard under Turing reductions. See Fig. 8 for an illustration. A full, detailed proof is provided in the Appendix. $\square$

Theorem 3.1 not only shows that the original result of (Otworowska et al. 2018) is robust with respect to the promise, but it has a further relevant implication: The conditions that are sufficient for ecological rationality to be possible (such as studied in the Ecological Rationality program) are not the same conditions that afford the tractable adaptation of ecological rationality. To guarantee tractability of the adaptation process itself, additional promises seem to be needed.

We next consider the effect of promising that the environment changes very slowly (i.e., only one situational change takes place at a time), and that for each change an ecologically rational toolbox still exists. In other words, it is promised that the toolbox can adapt from an initial (potentially very simple) environment and adjusts itself iteratively over time, slowly adapting to very small changes. We cast this conception of each step in such an iterative adaptation process in the form of the following input-output mapping.
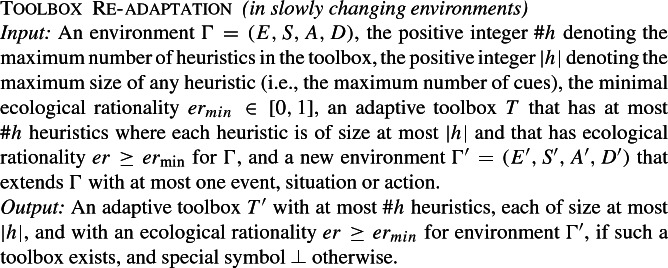


Note that in Toolbox Re-adaptation we start with an already previously adapted toolbox for a given environment $\Gamma$, and only ask that it be re-adapted for a new environment $\Gamma '$ that differs from $\Gamma$ in at most one event, situation or action. Given this strong constraint, one may expect the input-output mapping to be computationally much easier than adapting a toolbox for $\Gamma '$ scratch. Theorem 3.3 shows, however, that this is not the case, not even when it is promised that such an re-adaption is possible.

#### Theorem 3.3

Toolbox Re-adaptation is ${\mathsf {NP}}$-hard (under Turing reductions) even in a slowly changing environment. That is, Toolbox Re-adaptation is not tractable in a slowly changing environment, unless ${\mathsf {P}}={\mathsf {NP}}$. The problem remains hard when a promise is given that an ecologically rational toolbox exists.

#### Proof (sketch)

The general idea of the proof is the following. Suppose there is a polynomial-time algorithm *A* that solves Toolbox Re-adaptation in a slowly changing environment. Then we can use this algorithm *A* to construct a polynomial-time algorithm *B* that solves Toolbox Adaptation. The algorithm *B* works by iteratively constructing the environment $\Gamma$ event by event, action by action, and situation by situation, at each point using the algorithm *A* to compute and keep track of a toolbox that has high enough ecological rationality for the part of the environment constructed up to that point. By constructing the environment $\Gamma$ in an appropriate order, we can ensure that this procedure outputs an ecologically rational toolbox for $\Gamma$ (of the right size), if and only if one exists. This procedure also works for the case where we are given a promise that an ecologically rational toolbox (of the right size) exists. From this we can conclude that there exists no polynomial-time algorithm for Toolbox Re-adaptation in a slowly changing environment, unless one exists for Toolbox Adaptation, which would imply that ${\mathsf {P}}={\mathsf {NP}}$. A full, detailed proof is provided in the Appendix. $\square$

So far, the complexity analyses show that intractability of toolbox adaptation is quite a robust property. We close this section by making two more observations: None of the intractability results seem to be driven by potential richness of the action repertoire (see Corollary 3.1), nor by the potential intricacies of the selector or the individual heuristics in the toolbox (Corollary 3.2). In sum, the computational complexity intrinsic in toolbox (re-)adaptation seems to arise from the difficulty of finding a toolbox (however simple or complex) that has the right *fit* with the environment (even if a good fit is possible).

#### Corollary 3.1

Toolbox Adaptation is ${\mathsf {NP}}$-hard, even when restricted to the case where there are only two actions and in each situation exactly one action is satisfactory.

#### Proof (sketch)

The polynomial-time reduction that we use in the proof of Theorem 3.1 constructs only instances with two actions. Therefore, we get that the problem is ${\mathsf {NP}}$-hard also when restricted to the case where there are only two actions. $\square$

#### Corollary 3.2

Toolbox Adaptation is ${\mathsf {NP}}$-hard, even when restricted to the case where $\#h = 1$ or when restricted to the case where $|h| = 1$. That is, Toolbox Adaptation is ${\mathsf {NP}}$-hard even in the cases (i) where the selector heuristic is trivial (always selects the same heuristic), and (ii) where each heuristic is trivial (always selects the same action).

#### Proof (sketch)

The polynomial-time reduction that we use in the proof of Theorem 3.1 constructs only instances with one heuristic. Therefore, we get that the problem is ${\mathsf {NP}}$-hard also when restricted to the case (i) where the selector heuristic is trivial. For the case (ii) where each heuristic is trivial (but the selector heuristic is not), we can straightforwardly modify the reduction used in the proof of Theorem 3.1 to produce only instances where each heuristic is trivial. A more detailed proof is provided in the Appendix. $\square$

## Discussion

Our formal results significantly extend current understanding of the Adaptive Toolbox and the conditions under which toolboxes of heuristics can tractably be produced by adaptation processes. We have built on a finding by Otworowska et al. (2018) that the toolbox adaption problem is (Theorem 3.1), meaning that there cannot exist any tractable adaptation process that yields ecologically rational toolboxes. This result was proven relative to a formalization of the toolbox theory which is restricted to a specific set of heuristics known as ‘fast and frugal trees’ controlled by a selector operating by the same principles. But as explained in Sect. 2, this restriction is made for ease of exposition and without loss of generality (see also footnote 9). The intractability results for this restricted case rather underestimate the computational resources required for adapting toolboxes consisting of heuristics that are less constrained.

Besides its generality, the intractability result also proves to be quite robust against changes to the formalization that *prima facie* could be expected to affect it. For instance, one may intuit that if an environment is structured such that there exists a toolbox that is ecologically rational for that environment, then the problem of finding such a toolbox for that environment is tractable. Theorem 3.2 shows, however, that this intuition would be mistaken: even if it is promised that for the environment under consideration there exists a toolbox that meets the requirement of ecological rationality, it remains an problem to *find* that toolbox. In other words, the conditions that are sufficient for ecological rationality are not the same as the conditions that enable the tractable adaptation of ecologically rational toolboxes (an important distinction that we expand on later). Additional conditions will need to be imposed before the adaptation problem itself can be rendered tractable.

So what could the additional conditions be? Theorem 3.3 rules out another plausible condition for tractability: *viz.*, the condition that the environment changes slowly such that the adaptation process can gradually configure an ecological rational toolbox for the final environment via intermediate states of adaptation. Again, one may intuit that the intractability results established in Theorems 3.1 and 3.2 vanish when this condition is imposed. Nonetheless, Theorem 3.3 shows that this intuition is not correct: it is as intractable to adapt in slowly changing environments as in drastically changing environments, and this holds even if for each minor change in the environment there exists a toolbox which would preserve ecological rationality. In sum, appealing to the fact that our adaptive toolboxes would have evolved along with our environment over a long period of time does not solve the problem.

This last theorem (Theorem 3.3) also gives a deeper insight into what seems to be the core of the problem in toolbox adaptation. The fact that the environment may exhibit only small local changes does not ensure that toolboxes can be re-adapted with similarly small local changes. Instead, small local changes in the environment can necessitate global changes to the toolbox’s configuration to maintain ecological rationality. This means that ‘hill climbing’ adaptation procedures (Johnson et al. 1988; Newell and Simon 1972; Russell and Norvig 2003), or any other heuristics for adaptation [e.g. Davis (1991)], would get the adaptation process stuck at local optima, where the degree of ecological rationality can be arbitrarily off, not just compared to the global optimum, but in relation to any minimal rationality criterion. The observation reveals an apparent paradox for the Ecological Rationality program: Ecological rationality is itself a global property that is exceptionally sensitive to changes in an environment, underscoring moreover the importance of continuous adaptation in order to maintain it. But, as Theorems 3.1–3.3 show, maintaining it even in the face of small changes is intractable.

We would like to remind the reader that despite the great challenge we have presented, the challenge is presented so that it can be overcome, and not to dissuade anyone from pursuing a fascinating research program. Accordingly, we have constructive proposals for addressing the intractability challenge. Before presenting them, however, we turn to a set of objections we envision at this point, to ensure that the nature and significance of the challenge itself are completely clear.

### Objections

Our mathematical observations, though sound, could still be of limited significance if we made unnatural or irrelevant assumptions in our formal analyses of the toolbox adaption problem. It is therefore important to self-critically assess whether or not we have misplaced the problem. We consider possible objections to our formal treatment and explain to what extent they affect either the formal results themselves or their applicability.

**The Threshold Objection** *A threshold criterion for minimal ecological rationality in the formalization is unnatural and does not match how proponents of the research program conceive of it. They have not argued for either a particular threshold or the existence thereof.*

Toolbox theorists reject the idea that real-world, adaptive rationality is defined by optimality, because on their view,

however adaptive the behavior of organisms in learning and choice situations, this adaptiveness falls far short of the ideal of “maximizing” postulated in economic theory. Evidently, organisms adapt well enough to “satisfice”; they do not, in general, “optimize” (Simon 1956, p. 129).

Therefore, in our formalization, we have endeavored to avoid any optimality assumptions or appeal to ‘perfection’ which would require so-called ‘Laplacian demons’ (see e.g. Gigerenzer and Goldstein 1996, p. 3). In the formalism, it is not the case that a toolbox is rational only if all the actions it selects for in a given environment are optimal or perfect. On the contrary, we build in non-optimality in two ways. Firstly, in how we code actions as satisfactory or not for a given situation: the formalism leaves what would count as ‘satisfactory’ open to interpretation. The computational complexity analysis is independent of and insensitive to the semantics of ‘satisfactory’ here. Secondly, a toolbox need not always select satisfactory actions in order to count as ‘ecologically rational.’ It could fail to do so on many occasions and still be considered ecologically rational. All that is required is that there is some lower bound on how many satisfactory actions it picks, averaged over all possible situations it may encounter.

Yet one may object—so the Threshold objection goes—that building in this minimal ecological rationality criterion isn’t in the spirit of the theory. The originators see ecological rationality as a fit between our toolbox of heuristics and the environment. And the study of ecological rationality is to understand the nature of that fit: which heuristics work when, for which environments. Nowhere does the theory specify that ecological rationality would imply a minimal rational performance.

Our response is that this does not seem to invalidate our formalism, but rather shows a perhaps-unrecognized and unexplored commitment of Ecological Rationality, understood as an account of *rationality* specifically. Without any minimal threshold, anything goes. Even an agent who never picks any satisfactory actions for any situation would count as (ecologically) rational. This would make the notion of rationality void, in our opinion. To counter that possibility, we impose a free-to-interpret, but otherwise fixed, minimal threshold for ecological rationality, $er_{min}$, which can be any number between 0 and 1, but excluding 0.

We realize that allowing the option that $er_{min} = 1$ risks that our intractability results could be driven solely by that limit case scenario. If so, one could object that optimally is still smuggled in. Something similar could be said if the results were driven by the inclusion of high thresholds. Yet, our proofs show that this is not the case: each theorem also holds for any $er_{min}$, with $0< er_{min} < 1$.

In other words, for the interpretation and implications of our formal results, one not need commit to any particular performance threshold for ecological rationality, and the threshold can be set fairly low. Surely there is some threshold for which everyone would agree that a toolbox failing to meet that threshold is not rational in any meaningful sense. Indeed, Ecological Rationality is championed on the basis of the idea that humans are largely and surprisingly successful through the clever application of heuristics, so it would be strange to claim that human rationality consists in using heuristics to make arbitrarily poor choices and inferences.

**The Luck Objection** *That the adaptation process is intractable from a deterministic computation perspective is irrelevant. Adaptation processes are non-deterministic, and we clearly have evolved this type of rationality, so it seems to have just worked out.*

In our analyses we built on the classical theory of computational complexity (Arora and Barak 2009, see also Section 3.1). Consequently, in our analyses the notion of ‘computation’ is defined as a process that can in principle be simulated by a deterministic Turing machine. This foundation is less limiting then it may at first appear. results are known to have a straightforward and well-understood relation to probabilistic computation. For instance, even if we’d recast the adaptation problem as one where an ecologically rational toolbox is to be found in at least half of the situations (or in fact, any constant percentage), then the ${\mathsf {NP}}$-hardness results that we presented proves the impossibility of a tractable probabilistic computation process that meets that requirement (given another widely assumed conjecture, that ${\mathsf {P}}= \mathsf {BPP}$). In other words, the ability to tractably adapt ecologically rational toolboxes in a probabilistic sense would, just like the ability to tractably adapt ecologically rational toolboxes in a deterministic sense, imply that ${\mathsf {P}}= {\mathsf {NP}}$. To our knowledge, no toolbox theorists question the famous ${\mathsf {P}}\ne {\mathsf {NP}}$ conjecture. In fact, ${\mathsf {NP}}$-hardness and the ${\mathsf {P}}\ne {\mathsf {NP}}$ conjecture are widely referenced by toolbox theorists to point out the computational demons hidden in classical accounts of rationality (recall the quotation in 3.1 and see also Gigerenzer et al. 2011).

Be that as it may, the toolbox theorist may still object we’ve misconstrued the nature of the adaptation claim of ecologically rational toolboxes, and that there is no claim that evolutionary processes (phylogenetically or ontogenetically) would more or less reliably yield them, even probabilistically. The argument could go like this: So what that the adaptation process seems intractable? Clearly we have evolved this type of rationality (see also the Empiricist objection below), so it seems to have just worked out. Evolutionary process are not teleological, but in hindsight we can see that there have been (a concatenation) of lucky events that led us to have adapted ecologically rational toolboxes. This, however, seems to be begging the question. Adaptationist stories cannot appeal to a one-shot lucky event, but must appeal to a process that *tends* to bring about some property. Otherwise, it can only be claimed that the adaptation story is not impossible, not that it is a plausible explanation. Analogously, imagine that we learn that a coin has landed Tails on 100 consecutive tosses. It is possible that the coin was fair and that the sequence was the product of incredible luck. Without knowing in advance that the coin was fair and fairly flipped, however, we could not offer this as the *explanation* for the series of Tails. Similarly, we cannot be satisfied with the adaptation of a toolbox as the explanation for our rationality. Just as we would inquire whether the coin were constructed so that it could only land Tails, we must inquire whether some special feature of the context of adaptation would make the adaptation of an ecologically rational toolbox antecedently probable.

It is worth observing, furthermore, that it would not be enough for humans to have gotten lucky with an adaptive toolbox, despite the odds. This is because the environment, though stable enough for adaptation to be possible, does change. As our results show, re-adapting an already-ecologically rational toolbox in response to an individual small change is itself an intractable problem. So, essentially, an appeal to luck means “explaining” human cognition not just with one fluke series of Tails, but with many.

**The Empirical Success Objection** *The empirical evidence that people use heuristics—and that heuristics can perform spectacularly well—already justifies the conclusion that we have adapted this type of rationality. Formal analyses are irrelevant given the empirical evidence.*

This objection follows up on the Luck objection by suggesting that we have enough evidence about human cognition to infer that we did evolve an adaptive toolbox. We have lots of empirical evidence that humans and other animals use heuristics widely and successfully, so the lucky event (adaptation of an ecologically rational toolbox) must have taken place. We have two responses to this objection. First, it overstates the weight of the empirical evidence. Second, even if the empirical evidence were unequivocal, our results show that we would still lack a satisfactory explanation of how we came to have adaptive toolboxes.

The objection seems forceful because the examples of successful heuristic use can be so striking and compelling. Consider for instance the gaze heuristic: people and dogs alike catch flying objects with no thought at all, not by calculating trajectories, but by e.g. running so that their “gaze angle” stays constant (McLeod and Dienes 1996; Shaffer et al. 2004). If a heuristic can take the place of calculus here, what could heuristics not accomplish? From our set of examples of successful heuristic use, however, we cannot infer that human cognition is *as a rule* the product of heuristic use.^11^ This is a much stronger claim. The evidence is suggestive, but the examples still cover only a small minority of cognitive tasks and the set may be non-representative. So the empirical evidence is not yet sufficient to demonstrate the strong, general claim on which the objection relies.

Furthermore, it is not actually surprising that examples of heuristics performing well have been found. In the examples, both the task and the environment are chosen by the researchers, and it is known that if one is free to characterize the environment, there always exist tractable heuristics that perform well. Using human intelligence, it is not difficult to pair heuristics to environments and identify a task that the heuristic will do well at in that environment. This does not provide us with evidence for the separate claim that there exist adaptive processes which will tend to discover such pairings. The conditions sufficient for the existence of ecologically rational heuristics are not the same as the conditions which would make generating a toolbox of those heuristics tractable. For example, we could specify that our environment is such that being famous (for something) is highly correlated with winning competitions (for that something). If we set the task as guessing who is going to win such competitions, and specify that the right level of knowledge and the right options are present, then it is no surprise that ecological rationality is possible (that guessing that the famous person wins will be a very good strategy). The example is constructed for this to be the case. The existence of a strong correlation between fame and probability of winning (something) enables the heuristic to guess correctly, but it does not enable adaptation to find the heuristic. The two are separate issues, and the latter one is entirely neglected.

**The Just Wrong Objection** *Intractability is based on a notion of computability that just doesn’t fit with how cognition actually works. The formal analyses seem to be “just wrong,” as they allow for all kinds of free parameters, failing to model the specific heuristics that we, humans, have evolved.*

This objection expresses the possible intuition that there is just something wrong with our formal intractability analyses and how we approach the problem generally. This intuition may seem to be supported by the idea that all accounts face the same problem, or as Samuels put it (for cognition in general; cf. Otworowska et al. 2018, for rationality specifically):

(...) it is very widely assumed on inductive grounds by those who model cognitive processes that pretty much any interesting computational problem is superpolynomial in the worst case. Thus, the current criterion for intractability does little more than characterize those problems that are not of interest to a computational account of cognition (Samuels 2010, Ft. 4).

Reflecting Samuels’ complaint, we can provide a more precise interpretation of the intuition. Note that there are several other interpretations, the details of which call for different variants of our response here (for a fuller treatment, see van Rooij et al. 2019; van Rooij 2008).

In the context of the adaptive toolbox, the objection to the worst-case character of intractability results may go something like this: The formalization put forth and analyzed fails to model the specific problem that evolution solved in our (the human) case. Instead, the model and analyses contain many free parameters. It could well be the case that for some parameter settings, an adaptive toolbox is not tractably evolvable, but that these parameter settings represent ‘worst-case’ situations that may never arise in practice, and thus the intractability may be an artifact of overgeneralizing the input domain beyond ecologically relevant situations (see also van Rooij 2015).

This interpretation of our findings is not invalid, but does not invalidate the analyses. We fully agree that the results may be driven by unfortunate combinations of parameter settings, but draw an opposite conclusion from it. In fact, the point of (in)tractability analyses is to identify constraints on the input domain that render computations—which are intractable for unconstrained input domains—tractable (van Rooij et al. 2019; van Rooij 2008; van Rooij and Wareham 2008). The analysis is meant to be productive, revealing when theories are under-specified, so that the problematic parameter settings can be explicitly ruled out (such that the new worst-case parameter settings actually present a tractable problem). A theory for which this has been done is the stronger for it.

Furthermore, comparing the parameter settings for which competing theories are tractable provides possibilities for comparing the empirical plausibility of the theories. The Just Wrong Objection should seem much less compelling once it is recognized that it ought not remain the case that every theory of cognition is intractable. In fact, there is consensus that good theories of rationality must not be intractable (Fodor 1983, 2001; Lieder and Griffiths 2019; Martignon and Schmitt 1999), so as to not violate the ought-can principle (Oaksford and Chater 1998; Zenker 2017), and Adaptive Toolbox theorists have seen it as a virtue of their approach that their account meets this criterion, unlike competing, classical accounts of rationality (Gigerenzer 2008; Gigerenzer et al. 2008). For a program that aims specifically to give a tractable account of rationality, it is important to have formal tools to be able to assess whether and when it does. Our analyses demonstrate that the theory in current, unconstrained, form is not yet tractable. Formal analyses such as we have reported here can guide a research program aimed at developing tractable versions of the Adaptive Toolbox by pointing in the direction where tractable version may be found: they must lie in specifying the parameter settings.

### Lessons

We turn now to forward-looking lessons. For the Adaptive Toolbox, new research strategies should be used to ensure tractability. For naturalists seeking to ground normative claims in empirical ones, caution is required where it would not have been expected.

The current Ecological Rationality research program (which attempts to empirically validate the toolbox theory through examples of heuristics and experimental data) leaves the adaptation problem invisible. It also seems ill-suited for solving it directly, since it focuses on the application of heuristics from a pre-existing toolbox rather than on the adaptation of the toolbox itself, and the examples are handpicked. More constructively, then, our results demonstrate the need for a new sub-program within Ecological Rationality focused on identifying the constraints needed to ensure the tractability of the adaptation process specifically. This can only be achieved with the help of complexity analysis (as we explain in our response to the last objection, above); intuition is very misleading in this area. Fortunately, the systematic evaluation of possible constraints can also be very useful for the empirical part of the research program. Figuring out what would have enabled us to come to successfully apply heuristics could also tell us a great deal about how those heuristics work, which could in turn make it easier to populate the adaptive toolbox in a systematic way.

For epistemologists, this paper’s results shine the spotlight on a dependency of naturalism: the naturalist project relies on sufficiently developed and credible descriptive theories, since it must ground its normative claims in them. At bare minimum and even for moderate naturalists, it is quite important whether we in fact make most of our inferences using heuristics, just as it is quite important whether we are (or could be) Bayesian reasoners in some sense. Cognitive science is relatively young, however, and it is still not clear how human cognition is best explained. This is not an argument against naturalism itself, since it does not make non-naturalistic theories any more palatable. Instead, it is an argument for conscientiousness when drawing on scientific hypotheses, and for thinking about the implications of explanatory gaps for our normative projects. This requires, of course, staying abreast of what explanatory gaps there are.

One might have thought, in particular, that one could unproblematically build a naturalist, instrumentalist normative theory on the back of Ecological Rationality. The adaptive toolbox hypothesis is highly intuitive, the examples that have been provided are very compelling, and (in contrast to Bayesianism) it is easy to see how people can apply heuristics to draw inferences and so forth. The results presented here, however, show that we cannot yet be sure how far this research program can take us. We know that success can be achieved through heuristic use, but we cannot yet see how we could have evolved a full toolbox of heuristics for solving all of our problems. We may discover reasonable constraints that make toolbox adaptation tractable, or we may instead discover that only a part of human rationality stems from heuristic use. Our normative judgments cannot outpace our empirical understanding.

This is no reason for philosophers to stop incorporating the insights and discoveries that Ecological Rationality has brought us (some of which are independent of the adaptationist backstory anyhow). Nor do these results make Adaptive Toolbox less plausible or *worse off* than its competitors, which have their own tractability challenges (Kwisthout et al. 2011; van Rooij et al. 2018); they rather show that it does not have the tractability *advantage* that it appeared to have. On the flip side, this shows that the naturalist should not be too quick to abandon Bayesianism and the like as too-idealized and descriptively implausible. Just as all theories have tractability challenges, any and all have prospects for overcoming those challenges (for example, by using the methodology for determining the sources of intractability described in e.g. Blokpoel et al. 2013; van Rooij 2008; van Rooij and Wareham 2008; van Rooij et al. 2018).

Moreover, we can learn a great deal about what a good descriptive theory would look like by comparing Ecological Rationality and Bayesianism. The two sit at opposite extremes, with Ecological Rationality collecting concrete examples of successful heuristic application and Bayesianism providing an extremely abstract characterization of rational beliefs and choices. The contrast makes salient the weaknesses of both and the need for an intermediate offering. Bayesianism’s intractability problems result from its generality, and the possibility of re-interpreting almost everything as a Bayesian optimization means that it is hard to truly test and improve the theory. Ecological Rationality, in contrast, envies Bayesianism its all-encompassing abstraction. An improved adaptationist backstory would help to unify the plethora of examples and allow the prediction of new heuristics on theoretical grounds. In both cases, showing how tractability is possible is an important part of improving the theories’ plausibility and explanatory strength.

## Appendix

In this Appendix, we provide full proofs of the results discussed in the body of the paper. We restate the results before giving their proofs.

### Theorem 3.1

Toolbox Adaptation is , even when restricted to an arbitrary fixed $er_{min} \in (0,1]$.

### Proof

We give an alternative and simplified proof of the result by Otworowska et al. (2018). We firstly show hardness for the case of $er_{min} = 1$. Then we describe how the proof can be adapted for other $er_{min} \in (0,1]$.

We show ${\mathsf {NP}}$-hardness by giving a polynomial-time reduction from the ${\mathsf {NP}}$-complete problem of Dominating Set. In this problem, one is given an undirected graph $G = (V,F)$ and a positive integer *k*, and the question is to decide whether there exists a subset $D \subseteq V$ of at most *k* vertices in the graph that is a dominating set—that is, *D* is such that for all $v \in V$ it holds that either (i) $v \in V$ or (ii) there is some $v' \in D$ such that *v* and $v'$ are adjacent in *G*.

Let (*G*, *k*) be an input of Dominating Set, where $G = (V,F)$ is an undirected graph, *k* is a positive integer, $V = \{ v_1,\dotsc ,v_n \}$, and $F = \{ f_1,\dotsc ,f_m \}$. We construct an instance of Toolbox Adaptation as follows. We let $\#h = 1$ and $|h| = k$. Moreover, we let $er_{min} = 1$. We construct the environment $\Gamma = (E,S,A,D)$ as follows. As set of events, we take $E = \{ e_i\ |\ 1 \le i \le n \}$. The set $A = \{ a_1,a_2 \}$ of actions consists of two actions. As set of situations, we take $S = \{ s_i, t_i\ |\ 1 \le i \le n \}$. For each $1 \le i \le n$, we let the situation $s_i$ set exactly those events $e_j$ to true for which it holds that $i = j$ or $\{ v_j, v_i \} \in F$. Moreover, for each $1 \le i \le n$, we let the situation $t_i$ set all cues to false. We define the function *D* in such a way that in all situations $s_i$ only the action $a_1$ is satisfactory and in all situations $t_i$ only the action $a_2$ is satisfactory. This concludes our description of the construction of the instance of Toolbox Adaptation.

We argue that (*G*, *k*) is a yes-instance of Dominating Set if and only if the constructed instance is a yes-instance of Toolbox Adaptation.

$(\Rightarrow )$ Suppose that (*G*, *k*) is a yes-instance of Dominating Set. That is, there is some $D \subseteq V$ of size *k* that dominates all vertices in *G*. Let $D = \{ v_{j_1},\dotsc ,v_{j_k} \}$. We construct a toolbox that iteratively checks the events $e_{j_1},\dotsc ,e_{j_k}$. If any of them is true, the action $a_1$ is performed. Otherwise, the action $a_2$ is performed. Clearly, since *D* is a dominating set, this toolbox has an ecological rationality of $er_{min} = 1$. Moreover, since $|D| = k$, the toolbox fits the bounds given by $\#h$ and |*h*|.

$(\Leftarrow )$ Conversely, suppose that there exists a toolbox *T* that fits the bounds given by $\#h$ and |*h*| and that has ecological rationality $er_{min} = 1$. Due to the construction of $\Gamma$, it is straightforward to see that *T* must check *k* events $e_{j_1},\dotsc ,e_{j_k}$, and perform $a_1$ if and only if at least one of them is true. Since *T* has ecological rationality $er_{min} = 1$, we then know that $D = \{ v_{j_1},\dotsc ,v_{j_k} \}$ is a dominating set in *G*.

To modify the proof to work also for other $er_{min} \in (0,1]$, one can add an appropriate number of additional situations for which none of the actions is satisfactory. By doing this, one can decrease the ecological rationality $er_{min}$ that can maximally be attained by an arbitrary amount. Since $er_{min}$ is a fixed constant, this only adds a polynomial-time overhead to the reduction. $\square$

### Theorem 3.2

Toolbox Adaptation is ${\mathsf {NP}}$-hard (under Turing reductions) even when a promise is given that there exists an ecologically rational toolbox for the given environment (of the right size).

### Proof

Suppose that Toolbox Adaptation is tractable (i.e., solvable in polynomial time) when such a promise is given. We show that then SAT is solvable in polynomial time. (The polynomial-time algorithm for SAT that we specify is a polynomial-time Turing reduction from SAT to the variant of Toolbox Adaptation where a promise is given that there exists an ecologically rational toolbox of the right size for the given environment. Thus, this shows that the problem is ${\mathsf {NP}}$-hard under Turing reductions.)

Take an input $\varphi$ of SAT, i.e., $\varphi$ is a propositional formula. Due to the fact that Toolbox Adaptation is (Theorem 3.1), we know that there exists a polynomial-time reduction *R* from SAT to Toolbox Adaptation. We employ this reduction to construct an instance $R(\varphi )$ of Toolbox Adaptation in polynomial time. Moreover, this reduction *R* has the property that for any satisfiable formula $\varphi$, each solution for the input $R(\varphi )$ Toolbox Adaptation can be transformed in polynomial time (say with an algorithm $R'$) to a satisfying assignment for $\varphi$.

Now, due to our assumption that Toolbox Adaptation is tractable when an ecologically rational toolbox is promised to exist, we know that there exists an algorithm *A* that—for each input of Toolbox Adaptation for which there exists a solution—returns such a solution in polynomial time. We then run the algorithm *A* on the input $R(\varphi )$, leading to some output. If this output is not a toolbox, we return “unsatisfiable.” If this output is some toolbox, we run the algorithm $R'$ on the toolbox to get a truth assignment $\alpha$ for $\varphi$. If the algorithm $R'$ returns no truth assignment or if it returns a truth assignment $\alpha$ that does not satisfy $\varphi$ (we can check this in polynomial time), we return “unsatisfiable.” Otherwise, if it returns a satisfying truth assignment $\alpha$ for $\varphi$, we return “satisfiable.”

We claim that this algorithm correctly solves SAT in polynomial time. Suppose that $\varphi$ is satisfiable. Then $R(\varphi )$ is a yes-instance of Toolbox Adaptation. Thus, the algorithm *A* will output a valid solution (a toolbox) for $R(\varphi )$. Consequently, after running the algorithm $R'$ on this solution, we get a satisfying truth assignment $\alpha$ for $\varphi$, and thus the algorithm correctly outputs “satisfiable.” Conversely, if $\varphi$ is unsatisfiable, there does not exist any satisfying truth assignment for $\varphi$. Therefore, the algorithm will correctly output “unsatisfiable.” This concludes our proof. $\square$

### Theorem 3.3

Toolbox Re-adaptation is ${\mathsf {NP}}$-hard (under Turing reductions) even in a slowly changing environment. That is, Toolbox Re-adaptation is not tractable in a slowly changing environment, unless ${\mathsf {P}}={\mathsf {NP}}$. The problem remains hard when a promise is given that an ecologically rational toolbox exists.

### Proof

We give a polynomial-time Turing reduction from Toolbox Adaptation. By Theorem 3.2, this allows us to conclude the the problem of Toolbox Re-adaptation is ${\mathsf {NP}}$-hard under Turing reductions.

Take an instance $(\Gamma ,\#h,|h|,er_{\mathrm {min}})$ of Toolbox Adaptation, where $\Gamma = (E,S,A,D)$. Without loss of generality, we may assume that $\#h = 1$—because the reduction in the proof of Theorem 3.2 constructs only instances where $\#h = 1$. Moreover, let $n_E = |E|$ be the number of events, let $n_S = |S|$ be the number of situations, and let $n_A = |A|$ be the number of actions in $\Gamma$. We will solve this instance in polynomial time by querying an oracle for Toolbox Re-adaptation multiple times.

We firstly construct the environment $\Gamma _1$, consisting of a single event $e_1$, a single situation $s_1$, and a single action $a_1$ taken from $\Gamma$—in such a way that $\Gamma _1$ agrees with $\Gamma$, and such that the action $a_1$ is satisfactory in $s_1$. Moreover, we construct a toolbox $T_1$ that has ecological rationality $1 \ge er_{\mathrm {min}}$; this is easy, since $\Gamma _1$ has a single event, a single situation and a single action, and this action is satisfactory in the only situation.

Then, we proceed in $r = n_E + n_A + n_S - 3$ rounds. In each *i*-th round, we construct a toolbox $T_i$ with sufficient ecological rationality for some environment $\Gamma _i$ that consists of $\Gamma _{i-1}$ together with a new event, a new situation, or a new action from $\Gamma$, if possible. We do this by querying an oracle for Toolbox Re-adaptation several times. In the first $n_E - 1$ rounds, we iterate over all possible events from $\Gamma$ that do not appear in $\Gamma _{i-1}$. For each such event, the input that we give to the oracle consists of the environment $\Gamma _{i-1}$, the integers $\#h$ and |*h*|, the value $er_{\mathrm {min}}$, the toolbox $T_{i-1}$, and the environment $\Gamma '$ consisting of $\Gamma$ extended with the new event. If the oracle returns the special symbol $\bot$ for each possible event, situation and action, we terminate the entire algorithm and return $\bot$ also. Otherwise, the oracle returns some suitable toolbox $T_i$ for some pair $\Gamma '$. Then we let $\Gamma _i = \Gamma '$, and we continue with the next round.

In the next $n_A - 1$ rounds, we do the same for the actions appearing in $\Gamma$ but not in $\Gamma _i$. Then, in the last $n_S = 1$ rounds, we again do the same, but then for the situations appearing in $\Gamma$ but not in $\Gamma _i$.

After performing all rounds, we have either found a suitable toolbox $T = T_r$ that has ecological rationality $er \ge er_{\mathrm {min}}$ for the environment $\Gamma _r = \Gamma$, or we have terminated the procedure in some round and have returned $\bot$. We claim that the algorithm returns a suitable toolbox *T* (for the original instance of Toolbox Adaptation) if and only if such a toolbox exists.

$(\Rightarrow )$ Suppose that the algorithm returns a toolbox *T*. This can only happen when *T* is found in the *r*-th round. By the way we define the input to the oracle in the *r*-th round, we know that *T* must have at most $\#h$ heuristics, each of size at most |*h*|, and have ecological rationality $er \ge er_{\mathrm {min}}$ for $\Gamma _r = \Gamma$.

$(\Leftarrow )$ Conversely, suppose that a suitable toolbox *T* exists for $\Gamma = \Gamma _r$. Then we know that in each round, the oracle will return a suitable toolbox *T*. This is because we construct the rounds in such a way that we first add all events and actions, and then add all situations. Whenever we can add a new situation in such a way that a suitable toolbox exists that has high enough ecological rationality, we can safely do so (without having to backtrack later) because ecological rationality is monotone—meaning that adding a situation where a toolbox chooses a satisfactory action never decreases the ecological rationality of that toolbox. Therefore, after the *r*-th round, the algorithm will return some toolbox *T* for $\Gamma _r = \Gamma$ with at most $\#h$ heuristics, each of size at most |*h*|, and with ecological rationality $er \ge er_{\mathrm {min}}$ for $\Gamma$.

Since Toolbox Adaptation is ${\mathsf {NP}}$-hard, we can conclude that Toolbox Re-adaptation is ${\mathsf {NP}}$-hard under polynomial-time Turing reductions.

To see that the problem remains intractable even when a promise is given that an ecologically rational toolbox exists, it suffices to note that the proof of Theorem 3.2, also works when the polynomial-time algorithm for Toolbox Adaptation consists of the above algorithm combined with a polynomial-time algorithm for Toolbox Re-adaptation that is used to simulate the oracle queries. That is, there is no polynomial-time algorithm that solves Toolbox Re-adaptation for the case where a promise is given that an ecologically rational toolbox exists, unless ${\mathsf {P}}={\mathsf {NP}}$. $\square$

### Corollary 3.1

Toolbox Adaptation is ${\mathsf {NP}}$-hard, even when restricted to the case where there are only two actions and in each situation exactly one action is satisfactory.

### Proof

The polynomial-time reduction that we use in the proof of Theorem 3.1 constructs only instances with two actions. Therefore, we get that the problem is ${\mathsf {NP}}$-hard also when restricted to the case where there are only two actions. $\square$

### Corollary 3.2

Toolbox Adaptation is ${\mathsf {NP}}$-hard, even when restricted to the case where $\#h = 1$ or when restricted to the case where $|h| = 1$. That is, Toolbox Adaptation is ${\mathsf {NP}}$-hard even in the cases (i) where the selector heuristic is trivial (always selects the same heuristic), and (ii) where each heuristic is trivial (always selects the same action).

### Proof

The polynomial-time reduction that we use in the proof of Theorem 3.1 constructs only instances where $\#h = 1$. Therefore, we get that the problem is ${\mathsf {NP}}$-hard also when restricted to the case where $\#h = 1$.

For the other case, we can modify the proof of Theorem 3.1 as follows. In the reduction in the proof of Theorem 3.1, we construct the instance in such a way that $\#h = 1$ and $|h| = k$. In the modified reduction, we instead let $\#h = k$ and $|h| = 1$. The rest of the proof is entirely the same. The argument why the reduction is correct is entirely analogous. Thus, we get a polynomial-time reduction (showing ${\mathsf {NP}}$-hardness) that produces only instances where $|h| = 1$, and therefore we can conclude that the problem is ${\mathsf {NP}}$-hard also when restricted to the case where $|h| = 1$. $\square$

## Footnotes

1 The term ‘real world’ is of course not meant to rule out the good kind of abstraction away from unnecessary details, but rather the bad kind in which nothing concrete is actually abstracted *from*.

2 There are many epistemological positions that could be considered naturalistic to some degree, and we are not in any way aiming to reflect all of them in this task description. Our position is deeply naturalistic without giving up on normativity; this version of naturalism matches especially well with Ecological Rationality (our target psychological research program). A similar position is defended by Stich (1993), who sees the work of Herbert Simon—Ecological Rationality’s intellectual father—as particularly promising. More mainstream process reliabilism (as set forth in Goldman 1986, part I) is also structurally similar to Ecological Rationality, though importantly different in its insistence on truth as the primary good (a feature also discussed by Stich).

3 There is some controversy surrounding these results, regarding for example whether it makes sense to say that a heuristic can do better than optimization. See Parpart et al. (2018) and compare to Dana and Davis-Stober (2016). For our purposes, the important point is just that there are many real-world examples in which heuristics achieve truly excellent results, and using a more complicated procedure would not lead to improved choices or inferences (see Gigerenzer and Sturm 2012, for a summary of such examples).

4 We will refer throughout the paper to both the adaptive toolbox (theory or hypothesis) and to ecological rationality, which overlap to a great extent. ‘Ecological Rationality’ (capitalized) refers to the general program of research into judgment and decision making which is characterized by emphases on the use of simple heuristics, the importance of the specific environment, and the successes (rather than the failures) of agents. ‘Ecological rationality’ (uncapitalized) refers to the type of rationality studied by that research program. ‘Adaptive Toolbox’ refers to a more specific cluster of hypotheses, namely that human cognition is *as a rule* explained by the application of heuristics, that our ‘adaptive toolbox’ (uncapitalized) of heuristics is an adaptation, and that we are generally successful (ecologically rational) as a result of relying on this toolbox. Not all of Ecological Rationality depends on Adaptive Toolbox being true, but the latter makes the former much more compelling and interesting.

5 While the argument from adaptability is intuitive, it has been criticized; for example, Schulz (2011) questions whether the fact of adaptedness really speaks in favor of heuristics-based rather than classical rationality (see also Sterelny 2003; Polonioli 2015). We set these critiques aside in order to focus on our point, which we see as largely independent. The fact that there *are* such critiques, however, is certainly relevant, since it means that we cannot simply take for granted that humans have an adaptive toolbox; see also the Empirical Success Objection, in 4.1.

6 It is important to recognize that an appeal to as-if explanations does not avoid the problem either (van Rooij et al. 2018).

7 This is not to exclude other, (to epistemologists) less familiar competitors such as Predictive Processing (Clark 2013), which incidentally is in the same boat with respect to intractability (Otworowska 2018; Otworowska et al. 2014, 2018).

8 For example: “In many real-world situations ...Bayes’s rule and other ‘rational’ algorithms quickly become mathematically complex and computationally intractable, at least for ordinary human minds” (Gigerenzer 2000, p. 167).

9 This choice will be without loss of generality for our analyses in Sect. 3. The reason is threefold: (1) if adaptation is intractable for a special case (here fast and frugal trees) then it is also intractable for a more general case that includes this special case (see also Otworowska et al. 2018; 2) it is known that many heuristics can be formally rewritten as fast and frugal trees (Sweers 2015); and (3) the results we present in Sect. 3 hold also for all heuristics defined by some combination of 1) a search rule, 2) a stopping rule, 3) and a decision rule (which is the general template for heuristics as conceived by Adaptive Toolbox theory (see e.g. Gigerenzer and Sturm 2012; Martignon 2001), provided that each rule is indeed fast and frugal, i.e., does not use all the available information in the environment (*frugal*), and the rule processing time is limited by an upper bound (*fast*). Hence, for ease of presentation we can proceed with formalising heuristics as fast and frugal trees.

10 The computational problem Minimum Spanning Tree has an exponential-sized search space, but can be solved in polynomial time by exploiting structure in the space (see Cormen et al. 2009).

11 In fact, it is a point of discussion whether the empirical evidence favors heuristic use in general (Glöckner et al. 2014) or the lauded heuristics in particular (see e.g. Chater et al. 2003). For our purposes, we wish to remain neutral on these issues and focus on the question of whether the adaptive toolbox theory has the important explanatory virtue of avoiding the intractability problem.
